# Addressing the diversity of *Xylodon raduloides* complex through integrative taxonomy

**DOI:** 10.1186/s43008-019-0010-x

**Published:** 2019-07-02

**Authors:** Javier Fernández-López, M. Teresa Telleria, Margarita Dueñas, Andrew W. Wilson, Mahajabeen Padamsee, Peter K. Buchanan, Gregory M. Mueller, María P. Martín

**Affiliations:** 1 Departamento de Micología, Real Jardín Botánico-CSIC, Plaza de Murillo 2, 28014 Madrid, Spain; 2 Sam Mitchel Herbarium of Fungi, Denver Botanic Gardens, 909 York Street, Denver, CO 80206 USA; 3 Manaaki Whenua - Landcare Research, Auckland, 1142 New Zealand; 4 Chicago Botanic Garden, Plant Science and Conservation, 1000 Lake Cook Road, Glencoe, IL 60022 USA

**Keywords:** *Basidiomycota*, Biogeography, Corticioid fungi, Environmental niche, *Hymenochaetales*, Morphological traits, Multilocus phylogeny, New taxa

## Abstract

In this study, the taxonomic diversity of the *Xylodon raduloides* species complex (*Hymenochaetales*, *Basidiomycota*) is examined. Specimens were studied using an integrative taxonomic approach that includes molecular phylogenetic and morphological analyses, and environmental niche comparisons. Four different species were found inside the *Xylodon raduloides* complex, with a biogeographic distribution pattern bound by geographic regions: Europe, North America, Patagonia, and Australia–New Zealand. Molecular, morphological, and environmental evidences delimit two lineages within this complex: a Northern Hemisphere clade with longer basidiospores and wider ranges in temperature and precipitation tolerance, and a Southern Hemisphere clade with smaller and more spherical basidiospores, and an isothermal and more humid climate preference. The integrative taxonomic approach used in this study demonstrates congruence between data sets and shows how morphological and environmental characteristics contribute to the differentiation of fungal species complexes. By combining various sources of taxonomic information, three new species are described: *Xylodon laurentianus*, *X. novozelandicus,* and *X. patagonicus*.

## INTRODUCTION

Corticioid fungi represent a polyphyletic group delimited by effused and resupinate basidiomes that usually grow on dead wood. One descriptor for these fungi is “paint on wood” which accurately characterizes their thin crust of reproductive structures, which are among the most elementary in *Agaricomycetes*. Although traditional classification grouped these fungi in a single family, molecular phylogenetic analyses have identified up to 50 different families in at least 11 orders (Larsson 2007).

Studying the biodiversity of corticioid fungi presents an opportunity to explore their phylogeny. First, despite their apparent macromorphological homogeneity, according to Mueller et al. (2007), there are more than 1800 described species, making them a highly diverse group. Second, they have colonized a broad range of environments around the world (Hallenberg 1991). For these reasons, corticioid fungi offer unique opportunities to study speciation and the geographic patterns that result from this process.

The idea that fungi are free from dispersal barriers had a long tradition, so global distributions were accepted as normal (Lumbsch et al. 2008). Cosmopolitanism or a high similarity index in corticioid fungi distribution patterns have been reported in many studies (Gilbertson 1980, Ghobad-Nejhad 2011). However, molecular studies have demonstrated distinct biogeographic patterns related to hidden biodiversity (Taylor et al. 2006, Knight & Goddard 2015). In this context, the “everything is everywhere” hypothesis (Baas Becking 1934) for all fungal groups has given way to the argument that geographic range inferred for a fungal species strongly depends on the nature of the characters used for its delimitation (Taylor et al. 2000).

Species concepts in fungi remain an important discussion topic (Taylor et al. 2000, Öpik et al. 2016). The same species concept is not always applicable across all fungal taxa due to the multiple evolutionary processes that can lead to fungal speciation (e.g. horizontal gene transfer, hybridization, etc.; Giraud et al. 2008). During the last two decades, molecular tools have transformed the study of fungal biodiversity. Among all the regions tested in Schoch et al. (2012), the nuclear ribosomal internal transcribed spacer DNA region (ITS, the fungal barcode) in most cases has the highest resolving power for discrimination between closely related species. Even so, taxonomic/systematic studies benefit by including other genetic regions (Balasundaram et al. 2015). Fungal species concepts have evolved through time (Cai et al. 2011) and mycologists have benefited from the development of genealogical concordance phylogenetic species recognition (GCPSR) for describing fungal diversity (Taylor et al. 2000). Molecular data have provided a detailed view of previously hidden fungal diversity, enabling better use of traditional species recognition methods in morphology or mating compatibility to unmask this cryptic fungal diversity (Giraud et al. 2008). The implementation of GCPSR has revealed the presence of hidden diversity in several complexes of corticioid fungi where morphological species recognition approaches failed (e.g. *Serpula himantiodes,* Carlsen et al. 2011). However, with the increase of DNA regions used to estimate phylogenies, many researchers have argued the need to consider processes that could lead to discordance among gene phylogenies, that is, differences between gene-trees and species-trees (Edwards 2009; Heled & Drummond 2010). The use of different models, such as the coalescent theory (Kingman 1982) that allows gene tree heterogeneity, have been successfully applied for fungal species delimitation (e.g. *Hyphoderma paramacaronesicum,* Martín et al. 2018).

The search for evidence in addition to molecular phylogenetic data has emerged as a goal for species delimitation (Wiens 2007). Interest has increased in ecological traits as characters for species identification in many organisms (Rissler & Apodaca 2007). The combination of phylogenetics and niche modeling methodologies has proven useful in studying the mechanisms that shape biogeographic patterns (Raxworthy et al. 2007, Marske et al. 2012). With the development of GIS-based and cartographic approaches, the comparison of environmental niches has been proposed to study such evolutionary processes as sympatric speciation and niche conservatism (Warren et al. 2008, Broennimann et al. 2012, Ahmadzadeh et al. 2013). Due to the paucity of detectable macromorphological features in corticioid fungi, identification and comparison of environmental diagnostic traits could help in revealing their hidden diversity.

*Xylodon* (Pers.) Gray 1821 is a cosmopolitan white-rot fungus (*Hymenochaetales*, *Basidiomycota*), with an important role in ecosystem services due to their ability to alter wood structure and create habitat for other groups of organisms. As noted by Hibbett et al. (2014), it is one of the largest genera of wood-rotting fungi, with 162 current legitimate names (Robert et al. 2005; Robert et al. 2013). During the last ten years, six new *Xylodon* species have been described (Ariyawansa et al. 2015, Chen et al. 2018, Crous et al. 2018, Viner et al. 2018), also 59 combinations made (Hjortstam & Ryvarden 2009, Riebesehl & Langer 2017, Chen et al. 2018) and two new names were proposed (Hjortstam & Ryvarden 2009, Riebesehl & Langer 2017).

*Xylodon raduloides* (Riebesehl & Langer 2017), previously known as *Schizopora radula* (Hallenberg 1983), has been widely reported. It is widespread in Europe, and the Canary Islands (Hallenberg 1983, 1991, Langer 1994, Ryvarden & Gilbertson 1994, Melo et al. 2007, Ryvarden & Melo 2014), and is also known from North America (Hallenberg 1983, Langer 1994, Zhou et al. 2016), South America (Langer 1994, Greslebin & Rajchenberg 2003, Gorjón & Hallenberg 2013, Martínez & Nakasone 2014), temperate Asia (Langer 1994, Hallenberg 1983), and Australasia (McKenzie et al. 2000, Paulus et al. 2000).

In a broad phylogenetic study of the genus *Schizopora* (now included in *Xylodon*), some degree of genetic isolation between populations of *X*. *raduloides* was detected, also supported by intercompatibility studies (Paulus et al. 2000). The worldwide geographic distribution of *X*. *raduloides*, along with available molecular data, suggested it could be a species complex with the true diversity reflecting biogeography. In this study, the diversity and biogeographic relationships in *X*. *raduloides* are addressed using an integrative taxonomic approach (Dayrat 2005). Our aim is to achieve a comprehensive understanding of the taxonomic diversity of the complex through the use of multiple sources of evidence (multi-locus species coalescent phylogeny, morphological characters, and environmental equivalence analysis).

## MATERIALS AND METHODS

### Taxon sampling and morphological studies

A total of 39 vouchers of *Xylodon raduloides* (Table 1) were obtained from four fungaria (CFMR, MA-Fungi, NY, and PDD), cultures of the Forest Products Laboratory (USDA), and ICMP culture collection (World Data Center for Microorganism 2011). Specimens from Huinay (Los Lagos Region, Chile) were collected during fieldwork in 2013/2014. Specimens encompassed a broad geographic range (Europe, North America, Patagonia, and Australia–New Zealand regions), in order to better understand the internal diversity and biogeography of the *X*. *raduloides* complex.

**Table 1:** Specimens and sequences included in this study. Data of country and basidiospore size are shown if available. New sequences obtained in this study in bold

Species/specimens	Country	Basidiospore morphology			GenBank Accesion number			
		L	W	Q	ITS	LSU	*rpb2*	*tef-1α*
***X. raduloides*** Riebesehl & E. Langer								
NY s.n.	Cameroon	5.75	3.87	1.49	**KY962843**	–	–	–
FCUG 1972	Denmark	–	–	–	AF145568	–	–	–
MA-Fungi 70,457, 11074MD	France	5.33	3.37	1.58	**KY962827**	–	–	–
MA-Fungi 78,658, 11851IS	France	5.38	3.15	1.7	**KY962828**	–	–	–
MA-Fungi 79,314, 18336Tell.	France	5.03	3.3	1.52	**KY962830**	–	–	–
MA-Fungi 79,442, 12028IS	France	5.15	3.32	1.55	**KY962834**	–	–	–
FCUG 1055	Romania	–	–	–	AF145569	–	–	–
FCUG 2136	Spain (Canary Islands)	–	–	–	AF145565	–	–	–
MA-Fungi 608	Spain	5.11	3.02	1.69	**KY962826**	–	–	–
MA-Fungi 12,778, 2266MD	Spain	5.03	2.95	1.7	**KY962832**	–	–	–
MA-Fungi 12,864, 755MD	Spain	5.07	3.13	1.62	**KY962820**	–	–	–
MA-Fungi 12,877, 6996Tell.	Spain	5.25	2.75	1.91	**KY962821**	–	–	–
MA-Fungi 22,499, 4719MD	Spain	5.4	3.35	1.61	**KY962822**	**KY962861**	–	–
MA-Fungi 22,513, 4736MD	Spain	5.48	3.49	1.57	**KY962823**	**KY962862**	–	–
MA-Fungi 35,643	Spain (Canary Islands)	6	4	1.5	**KY962831**	**KY962858**	**KY967054**	–
MA-Fungi 75,130, GP2241	Spain	–	–	–	**KY962824**	**KY962863**	**KY967057**	**KY967079**
MA-Fungi 75,244, GP2162	Spain	5.3	3.3	1.6	**KY962833**	–	–	–
MA-Fungi 75,272, GP2253	Spain	6	4	1.5	**KY962829**	**KY962859**	**KY967053**	**KY967077**
MA-Fungi 75,310, GP2291	Spain	5.16	3.05	1.69	**KY962825**	**KY962864**	**KY967055**	**KY967080**
MA-Fungi 90,709, 002JFL	Spain	4.95	3.24	1.53	**KY962844**	**KY962860**	**KY967056**	**KY967078**
***X. laurentianus*** sp. nov.								
CFMR, DLL2009–049	USA (Minnesota)	5.34	3.06	1.74	–	**–**	–	**–**
FPL (USDA), ex-culture DLL2009–049	USA (Minnesota)	–	–	–	JQ673187	**KY962866**	–	**KY967075**
CFMR, DLL2009–082	USA (Minnesota)	–	–	–	JQ673188	–	–	–
CFMR, DLL2009–087	USA (Minnesota)	–	–	–	JQ673189	–	–	–
CFMR, HHB-719, holotype	USA (Washington DC)	5.1	3.09	1.65	–	–	–	–
FPL (USDA), ex-culture HHB-719	USA (Washington DC)	–	–	–	**KY962845**	**KY962865**	–	**KY967076**
***X. patagonicus*** sp. nov.								
ICMP 13832, ex-culture MR106	Argentina	–	–	–	AF145581	**KY962848**	**KY967058**	**KY967074**
MA-Fungi 90,702, 14,180 M	Chile	5.26	3.51	1.5	**KY962836**	**KY962854**	**KY967062**	–
MA-Fungi 90,703, 3567MPM	Chile	4.38	2.85	1.53	**KY962841**	–	–	–
MA-Fungi 90,704, 3341MPM	Chile	4.58	3.18	1.44	**KY962840**	–	**KY967060**	–
MA-Fungi 90,705, 14007MD	Chile	4.4	3.1	1.42	**KY962835**	–	**KY967063**	–
MA-Fungi 90,706, 19705Tell.	Chile	4.56	3.17	1.44	**KY962838**	**KY962856**	**KY967064**	–
MA-Fungi 90,707, 19684Tell., holotype	Chile	4.45	3.12	1.43	**KY962837**	**KY962855**	**KY967061**	–
MA-Fungi 90,708, 3340MPM	Chile	4.32	2.9	1.49	**KY962839**	**KY962857**	**KY967059**	–
***X. novozelandicus*** sp. nov.								
ICMP 13833, ex-culture PB 98/41	Australia	–	–	–	AF145580	**KY962853**	**KY967068**	**KY967073**
FCUG 678	Canada	–	–	–	AF145564	–	–	–
MA-Fungi 74,919, 12836IS	France	5.2	3.15	1.65	**KY962842**	–	–	–
ICMP 13829, ex-culture PB 97/153	New Zealand	–	–	–	AF145577	**KY962850**	**KY967067**	**KY967071**
PDD 70716, Paulus 98/81	New Zealand	4.74	3.3	1.44	–	–	–	–
ICMP 13841, ex-culture Paulus 98/81	New Zealand	–	–	–	AF145579	**KY962852**	**KY967065**	**KY967072**
PDD 70718, Paulus 98/20, holotype	New Zealand	3.77	2.8	1.35	–	–	–	–
ICMP 13838, ex-culture Paulus 98/20	New Zealand	–	–	–	AF145578	**KY962851**	–	**KY967069**
PDD 70720, Paulus 98/104	New Zealand	4.52	3.3	1.37	–	–	–	–
ICMP 13840, ex-culture Paulus 98/104	New Zealand	–	–	–	AF145576	**KY962849**	**KY967066**	**KY967070**
PDD 91616	New Zealand	5.34	3.48	1.53	GQ411525	–	–	–
***X. flaviporus*** (outgroup)								
MA-Fungi 79,440, 12094IS	Germany	–	–	–	**MH260071**	**MH260066**	**MH259319**	**MH758542**

The initials MD, MPM and Tell. correspond to M. Dueñas, M.P. Martín and M.T. Telleria respectively

Colours of dried basidiomata follow the ISCC-NBS *Centroid Color Charts* (Kelly & Judd 1976). Measurements and drawings were made from microscopic sections mounted in 3% aqueous KOH and Congo Red solution and examined at magnifications up to 1250× using an Olympus BX51 microscope. The length (L) and width (W) of 20 spores and 10 basidia were measured from each specimen. Mean values and length/width ratios (Q) for each spore were calculated (Table 1). Line drawings were made with a Leica DM2500 microscope with the aid of a drawing tube.

### DNA extraction, amplification and sequencing

DNA isolation, amplification, purification and sequencing of four loci (ITS nrDNA, LSU nrDNA, *tef*-1α, *rpb*2) were performed following Martín et al. (2018). The raw sequences generated were edited, and contigs were assembled using Geneious version 9.0.2 (Kearse et al. 2012). Consensus sequences were accessioned in the EMBL/GenBank/DDBJ databases, and accession numbers are presented in Table 1.

Evaluation of EMBL/GenBank/DDBJ databases for *X*. *raduloides* sequence data was performed to maximize the molecular information available for this taxon. One sequence from *Xylodon flaviporus* (Riebesehl & Langer 2017) per marker was added to each dataset as an outgroup in phylogenetic analyses. MAFFT (Katoh & Standley 2013) was used to obtain sequence alignment for each region, with additional evaluation and edits of alignment performed using Geneious. A fifth alignment was performed for combined ITS + LSU sequence data. For this dataset, samples that lacked the LSU sequence were given a string of ‘?’ to represent missing data.

### Phylogenetic analyses

Combined ITS + LSU phylogenetic tree estimation was performed using Bayesian inference (BI) implemented in BEAST v2.4.3 (Drummond & Rambaut 2007, Bouckaert et al. 2014). Site model partition was defined using BEAUti v2.4.3 interface for ITS and LSU separately. HKY + G substitution model was selected for both, as the closest available in BEAST from the results obtained in jModelTest2 (Darriba et al. 2012) following Bayesian information criterion (BIC). We used relative timing with an uncorrelated lognormal relaxed clock by calibrating the tree with a value of 1 in the root for the *X*. *raduloides* clade (Drummond et al. 2006). Coalescent (constant sites) model was used as tree prior. Two MCMC runs were specified for 50 million generations, sampling every 5000th generation. Tree and log files were combined in Logcombiner v.1.7 and results were visualized in Tracer v.1.6 (Rambaut et al. 2018), to evaluate whether the effective sample size (ESS) values were above 200. The resulting trees were summarized in a maximum clade credibility tree by TreeAnnotator v.1.7. with a burn-in of 5000. The same procedure was used to separately estimate phylogenetic trees for each region (ITS and LSU).

**Fig. 1:**
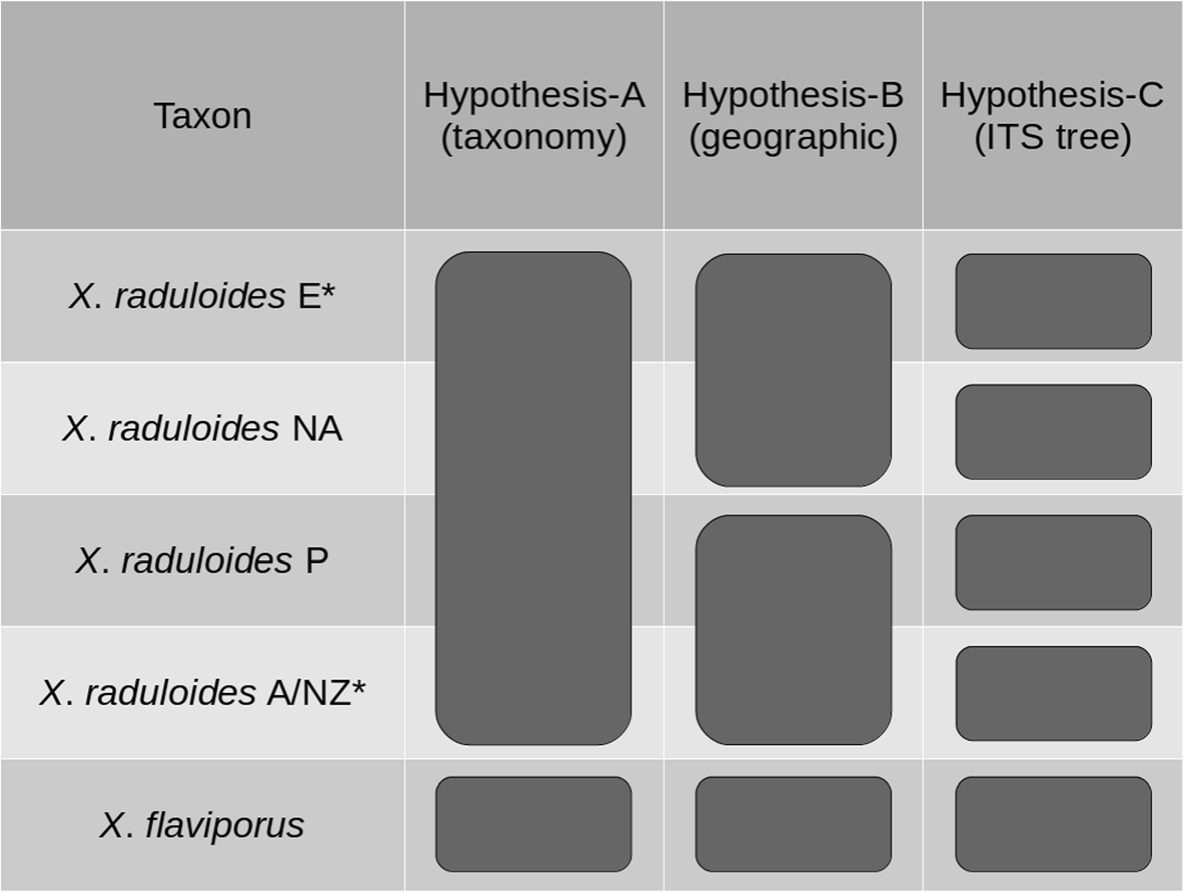
Hypothesized specimen assignment to each species hypothesis-model tested in this study. Species delimitation models are shown as columns, while lineages are shown as rows. Letters indicate geographic regions: E = Europe; NA = North America; P = Patagonia; A/NZ = Australia–New Zealand. (*) In Model-C, European taxon includes one sample from Cameroon (NY s.n.) and Australia–New Zealand taxa includes one specimen each from France (MA-Fungi 74,919) and Canada (FCUG 678)

A multi-locus species coalescent approach was used to test alternative species delimitation hypotheses (Grummer et al. 2014). To accomplish this, a competing species delimitation model was used (Fig. 1). We explored a priori assignment of individuals to lineages following three hypotheses based on different sources of information. The first hypothesis (hypothesis-A) addressed traditional taxonomic classification, including all *X*. *raduloides* specimens as a single species, separated from *X*. *flaviporus* as outgroup. In the second hypothesis (hypothesis-B) geospatial characters were used by grouping specimens according to a North-South distribution, including all specimens from the Northern Hemisphere as a first species and assigning all specimens from the Southern Hemisphere as a second species. Finally, a DNA barcoding species delimitation obtained from the ITS tree was addressed in the third hypothesis (hypothesis-C), assigning each specimen to the species defined by the ITS tree.

We estimated species trees for each model using the coalescent-based inference program *BEAST with the four amplified DNA regions (ITS, LSU, *tef*-1α and *rpb*2). This method co-estimates gene and species trees from sequence data taking into account evolutionary processes that could generate species-tree/gene-tree discordance, such as incomplete lineage sorting (Mallo & Posada 2016). Substitution and clock models for each region were selected in the same procedure as for the ITS + LSU tree. Constant population function (population mean = 1) was used to model the species tree population size. A coalescent constant population prior was used to build the species tree. Tree and log files were managed in the same way as for the ITS + LSU tree (ESS values above 200; burn-in = 5000). In order to visualize the species tree and concordance between the four DNA regions, the Densitree v2.01 package included in BEAST v2.4.3 was used (Bouckaert 2010).

To assess the suitability of each species delimitation hypothesis proposed, Bayes factor delimitation (BFD) was performed following the framework of Grummer et al. (2014). Marginal likelihood for each hypothesis (MLEs), measured as log likelihoods, are calculated from the Bayesian posterior distributions through stepping-stone analyses using Path Sampler Analyser (BEAST model-selection package version 1.0.2; default parameters: alpha = 0.3, steps = 24, chain length = 100,000 and burn-in = 50%). Bayes factors are calculated as two times the difference in MLE between the best-fitting and alternative hypothesis (2lnBf). Hypothesis-C was used as the standard to compare against the other two hypotheses (hypothesis-C vs hypothesis-A and hypothesis-C vs hypothesis-B). Values of 2lnBf between 0 and 2 are interpreted as no significant differences in support for the two hypotheses. Values over 10 means decisive support in favor of the best-fitting hypothesis over its alternative (Kass & Raftery 1995).

### Statistical tests of morphological characters

Basidiospore morphology was analyzed since sexual structures are valuable for species differentiation in fungi. One-way ANOVA tests were performed to assess the significance of differences in spore morphology between clades identified from multi-locus species coalescent analyses. Differences in spore morphology were evaluated using a Tukey HSD post-hoc test. Exploratory plots (i.e. residuals vs fitted values, normal Q-Q plots and residuals vs leverage) were used to detect and remove outliers from the morphological dataset. Two specimens (MA-Fungi 90,702 and MA-Fungi 74,919) from the total of 30 in the morphological dataset were removed after outlier identification.

### Environmental niche equivalence analyses

We examined environmental characteristics to assess the degree of niche equivalency between species delimited with multi-locus species coalescent analyses. For each of the studied specimens (out-group excluded), geographical location (longitude and latitude) was obtained from herbarium labels or collection information. A set of 19 bioclimatic variables related to temperature and precipitation were obtained from the WorldClim2 cartographic dataset (Fick & Hijmans 2017, Table 2).

**Table 2:** Description of bioclimatic variables used in environmental niche modeling from WorldClim2

Name	Variable
BIO1	Annual Mean Temperature
BIO2	Mean Diurnal Range (Mean of monthly (maxtemp-mintemp))
BIO3	Isothermality (BIO2/BIO7)(*100)
BIO4	Temperature Seasonality (standard deviation * 100)
BIO5	Max Temperature of Warmest Month
BIO6	Min Temperature of Coldest Month
BIO7	Temperature Annual Range (BIO5-BIO6)
BIO8	Mean Temperature of Wettest Quarter
BIO9	Mean Temperature of Driest Quarter
BIO10	Mean Temperature of Warmest Quarter
BIO11	Mean Temperature of Coldest Quarter
BIO12	Annual Precipitation
BIO13	Precipitation of Wettest Month
BIO14	Precipitation of Driest Month
BIO15	Precipitation Seasonality (Coefficient Variation)
BIO16	Precipitation of Wettest Quarter
BIO17	Precipitation of Driest Quarter
BIO18	Precipitation of Warmest Quarter
BIO19	Precipitation of Coldest Quarter

* means multiplication, those variables are multiplied by 100 to avoid decimals

To characterize the bioclimatic niche for each species, a total of 10,000 random points was generated over the entire study area (worldwide) and their bioclimatic features were extracted from the 19 variables. This dataset, in addition to bioclimatic values for specimen locations, was used to calibrate a Principal Components Analysis (PCA) that represents the environmental space. Then, a kernel density function was applied to obtain the smoothed density of species occurrences in this environmental space. Further niche overlap analyses were performed using these kernel distributions rather than actual species occurrences (Broennimann et al. 2012).

In order to assess whether environmental niche can be used as a diagnostic character to distinguish between species of the *X*. *raduloides* complex, equivalence tests were performed using Schoener’s D index (Schoener 1970). This index is a metric of ecological similarity that ranges from 0 (no niche overlap) to 1 (complete overlap). Equivalence tests were conducted by comparing the environmental overlap values (D) of pairs of *Xylodon* species to a null distribution of 100 randomized overlap values. We determined non-equivalence of environmental niches if the niche overlap value of the two species being compared differed significantly from the overlap values from the null distribution. All analyses were performed in the R platform (R Development Core Team, 2014) using “ecospat” R package v1.0 (Di Cola et al. 2017).

## RESULTS

### DNA extraction, amplification and sequencing

A total of 77 sequences were generated in this study: 27 sequences for ITS region, 20 for LSU, 17 for *rpb*2 and 13 for *tef*-1α (Table 1). The maximum lengths of sequences were 618 for ITS, 1347 for LSU, 884 for *rpb*2 and 748 for *tef*-1α. The final alignments, including sequences retrieved from the EMBL/GenBank/DDBJ databases contained 41 ITS sequences for a dataset length of 502 characters, 20 LSU sequences with 772 characters, 17 *rpb*2 sequences with 646 characters and 13 *tef*-1α sequences with 613 characters. No *X*. *raduloides* sequences were available for *rpb*2 and *tef*-1α regions from the EMBL/GenBank/DDBJ databases.

### Phylogenetic analysis

**Fig. 2:**
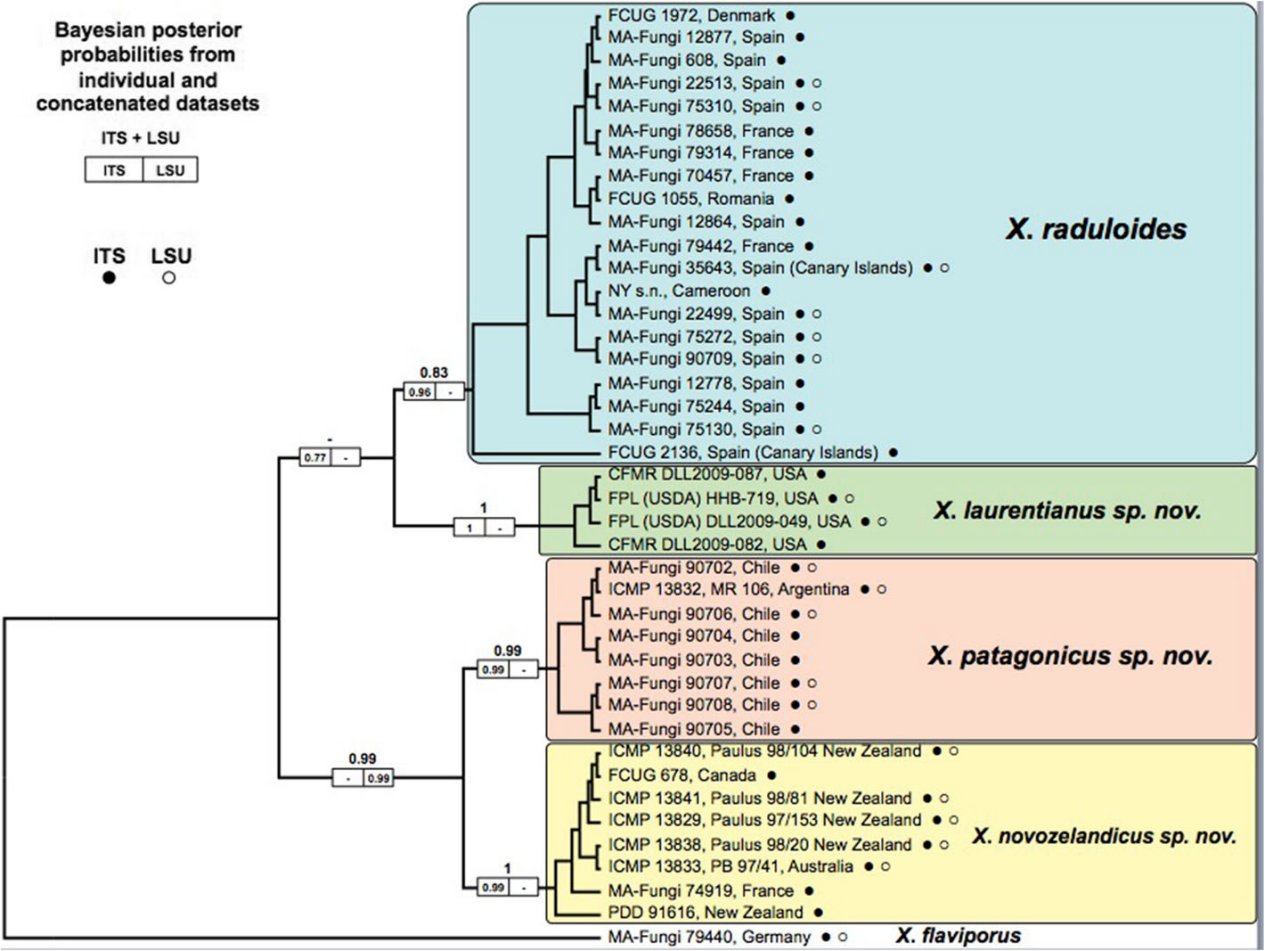
Topology of ITS + LSU tree obtained by Bayesian inference using BEAST. Bayesian posterior probabilities for the combined ITS and LSU dataset are indicated in the larger number above the boxes. Individual gene posterior probabilities for ITS and LSU regions are indicated in the left and right boxes on a branch, respectively. Filled and empty circles indicate whether ITS or LSU sequence of a sample was used in the analysis

Results of the phylogenetic analysis of ITS, LSU, and ITS + LSU alignments are summarized in Fig. 2. All effective sample sizes were higher than 200 for all parameters. Bayesian inference analyses suggest the division of *Xylodon raduloides* complex into four well-supported monophyletic clades (posterior probabilities (PP) ≥ 0.96 for all clades in the ITS tree), each restricted to their geographical distribution: Europe, North America, Patagonia, and Australia–New Zealand (Fig. 2). The only exceptions to this strong geographic pattern were two specimens in the Australia–New Zealand molecular clade that came from Europe and North America, while one specimen from Africa was resolved within the European clade.

**Fig. 3:**
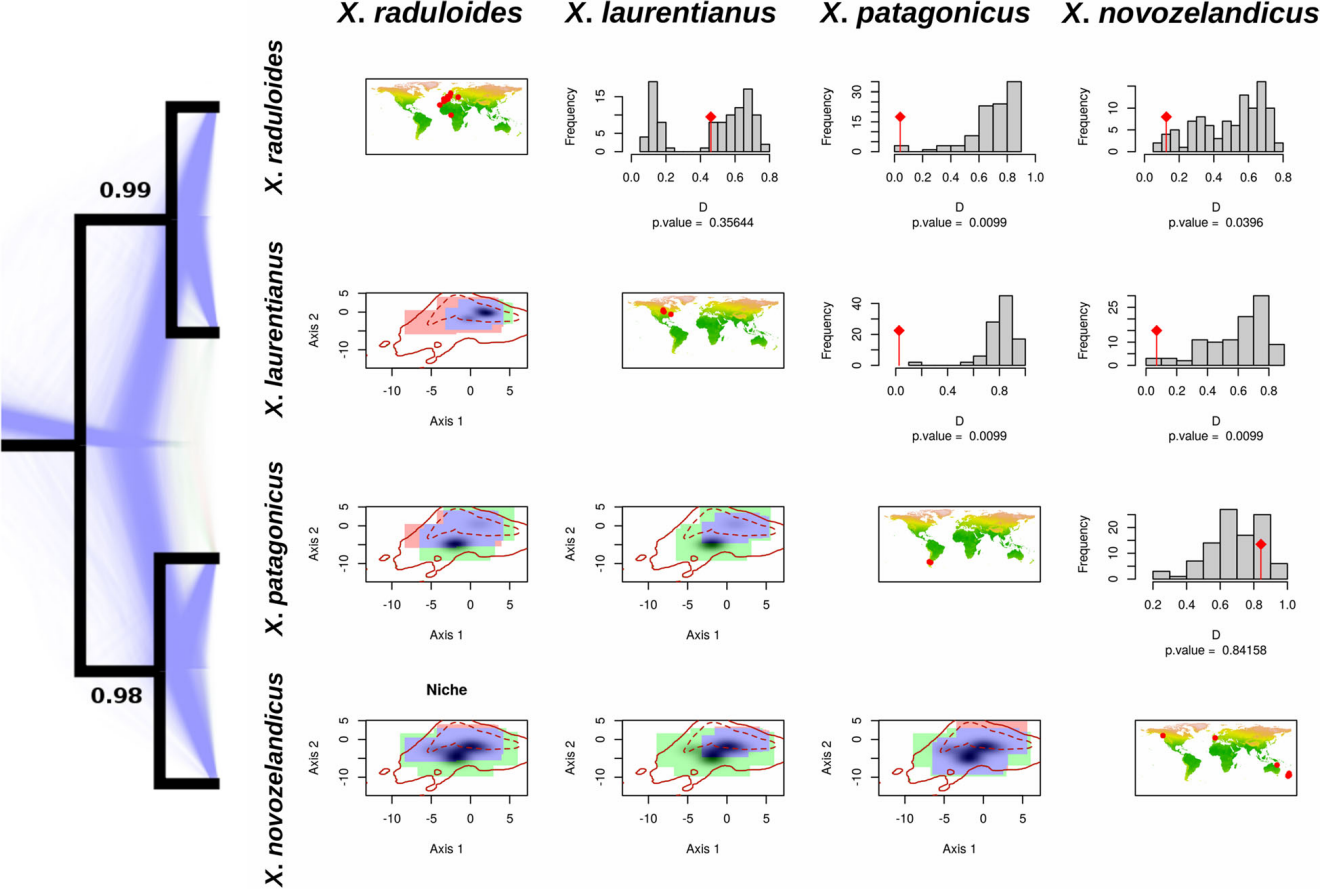
Species tree obtained from *BEAST applying multi-locus species coalescent approach for the four DNA regions used in the analyses (ITS, LSU, *rpb*2 and *tef*-1α) and results from niche overlap analyses. Tree topology represents Model-C obtained from the ITS analyses. Posterior probabilities are shown as supports for each node. Blue background trees represent bootstrap gene tree topologies obtained from *BEAST. Diagonal maps show locations of the specimens. The upper right triangle of the matrix represents the results from niche overlap analyses. *P*-values of the equivalence test are shown above each histogram of randomization procedures. The bottom left triangle of the matrix shows species niches resulting from PCA analysis. The blue areas show overlapping environmental niches, while green and red represent niche ranges for row and column species, respectively. Shaded areas indicate kernel distributions for row species. Solid and dotted lines represent environmental availability for the whole study area

Marginal likelihoods from multi-locus species coalescent analyses for each hypothesis and Bayes factors for hypothesis comparisons are shown in Table 3. The species hypothesis provided by the ITS barcoding approach (hypothesis-C) was the most probable scenario following Bayes factor scores (hypothesis-C vs hypothesis-A 2lnBf = 78.88; hypothesis-C vs hypothesis-B 2lnBf = 22.24), obtaining a Bayes factor > 10 for the second more probable scenario (“decisive” support for hypothesis-C against hypothesis-B). The species tree obtained from the multi-locus species coalescent approach for hypothesis-C defined four species divided into two well-supported clades (PP > 0.98; Fig. 3). The first clade grouped the two Holarctic species (European and North American species) and the second clade included Antarctic-Australian species (Patagonian and Australian–New Zealand species). The MCMC sample of gene trees obtained from *BEAST analyses for hypothesis-C was visualized by Densitree v2.01 and showed a high level of genealogical concordance with single DNA region trees (Fig. 3).

**Table 3:** Marginal likelihood estimates from each species tree hypothesis and Bayes factors (2lnBf) for hypotheses comparisons

MLE		2lnBf	
Hypothesis–A	− 5471.95	Hypothesis–C vs Hypothesis–A	78.88
Hypothesis–B	− 5442.63	Hypothesis–C vs Hypothesis–B	20.24
Hypothesis–C	− 5432.51

### Statistical tests of morphological characters

**Fig. 4:**
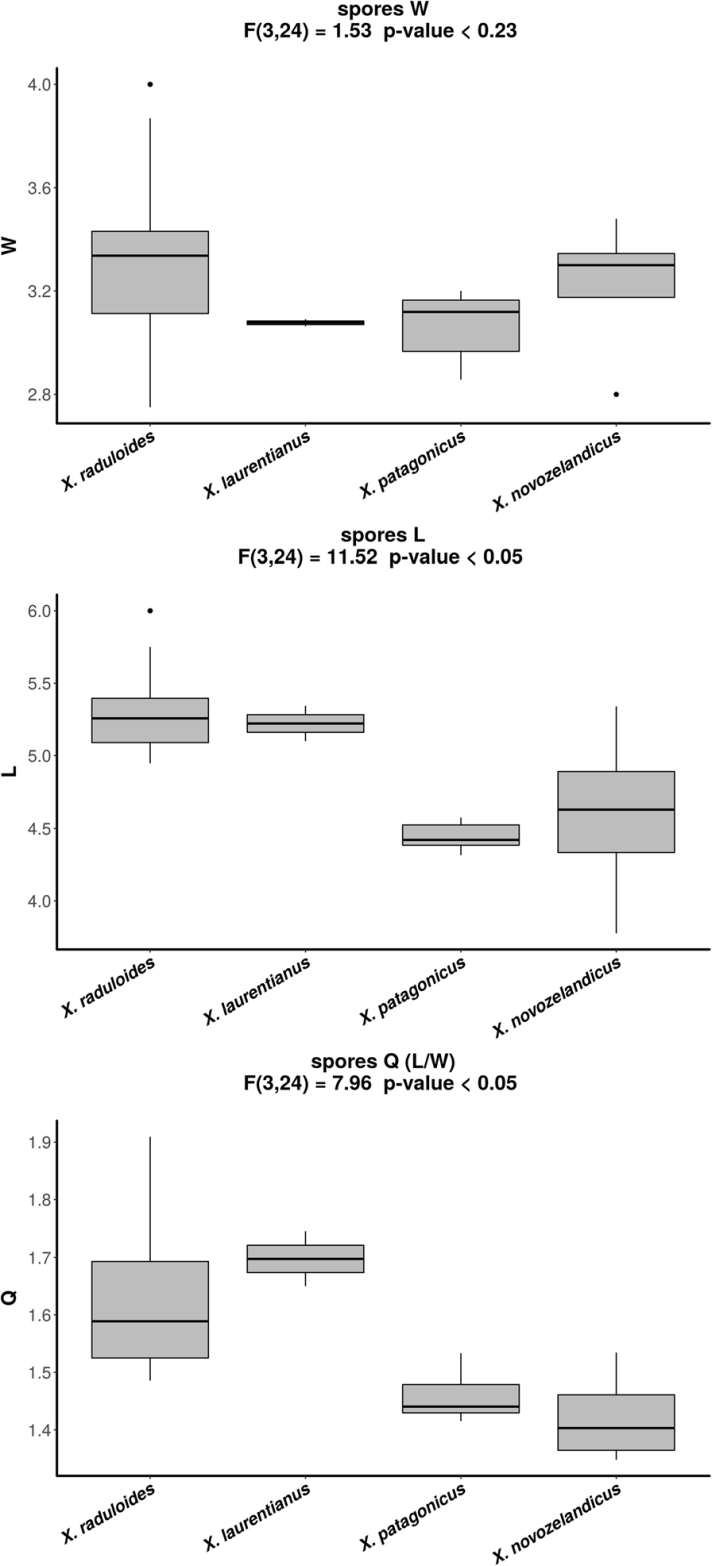
ANOVA results for basidiospore morphology. Bar plots of *Xylodon* species basidiospore width (W), length (L) and length/width ratios (Q)

ANOVA on basidiospore width, length and length/width ratio was conducted on 15 European specimens; one sample from Cameroon (NY s.n.); two North American specimens; six Patagonian specimens; and four Australia–New Zealand specimens (Fig. 4). The analysis did not detect any difference in spore width between species (F(3, 24) = 1.53, *P*-value = 0.23). However, differences were detected in spore length and length/width ratios (Q) between Northern (Europe and North America) and Southern (Patagonia and Australia-New Zealand) hemisphere samples (F(3, 24) = 11.52, *P*-value < 0.05 and F(3, 24) = 7.96, *P*-value < 0.05 respectively; Fig. 4). In the post-hoc Tukey HSD tests significant differences were found for spore length/width ratios between inter-hemisphere comparisons: Europe and Patagonia; Europe and Australia-New Zealand; North America and Patagonia; and North America and Australia–New Zealand (*P*-values < 0.05). However, the Tukey HSD test did not show significant differences in spore length in the inter-hemisphere comparison between North America and Australia–New Zealand (*P*-value = 0.19).

### Environmental niche equivalency analyses

The PCA performed with the 19 bioclimatic variables accumulated 76.71% of the variance in the first two axes (Axis 1: 50.32%; Axis 2: 26.39%; Fig. 3). For niche comparisons, two axes rather than one were selected to obtain a more complete view of niche dimensions, since the first axis represented only 50% of environmental variability. Axis 1 described mainly a gradient between regions with high temperature seasonality (BIO4) and high temperature annual range (BIO7) (positive values in axis 1) and areas with a high isothermality (BIO3) (negative values in axis 1). The environmental pattern of Axis 2 was less clear with areas of high temperature diurnal range (BIO2) and high precipitation seasonality (BIO15) on one side (positive values in axis 2) and regions with high precipitation during dry seasons (BIO14, BIO17) on the other side (negative values in axis 2). Kernel densities were then built for each clade from the coordinates (PC scores) of group occurrences in this two-dimensional environmental space created by the two first axes of the principal components analyses.

In general, environmental space occupied by North American and European occurrence kernels were placed in higher values for both axis 1 and axis 2 in the PCA (top-right) than Patagonian and Australia–New Zealand kernels (bottom-left, Fig. 3). That pattern describes a more seasonal niche for Northern Hemisphere species, while Southern Hemisphere species showed preferences for isothermal and humid areas.

Niche equivalence could not be rejected between North American and European species (*P*-value = 0.35, Fig. 3). A similar pattern was found for Patagonian and Australia–New Zealand niches, where the niche equivalence hypothesis could not be rejected between these two species (P-value = 0.84, Fig. 3). However, when inter-hemisphere environmental niches were compared, significant differences were observed for all species (niche equivalence was rejected; *P*-values < 0.05, Fig. 3).

Based on a combination of taxonomic information, three new species are described here: *Xylodon laurentianus*, *X. novozelandicus,* and *X. patagonicus*.

## TAXONOMY

**Xylodon raduloides** Riebesehl & Langer, *Mycol. Progr*. **16**: 649 (2017).

*Replaced name: Poria radula* Pers., *Observ. Mycol*. **2**: 14 (1800).

*Synonyms: Polyporus radula* (Pers.) Fr., *Syst. Mycol*. **1**: 383 (1821); nom. Sanct.

*Schizopora radula* (Pers.) Hallenb., *Mycotaxon* **18**: 308 (1983).

*Hyphodontia radula* (Pers.) Langer & Vesterh., *Nordic J. Bot.* **16**: 212 (1996).

*Kneiffiella radula* (Pers.) Zmitr. & Malysheva, *Pyatnadts. Respubl. Molod. Nauchn. Konf.*: 103 (2004).

Non *Xylodon radula* (Fr.) Tura et al. 2011*, Biodiv. Heterobasid. non-gilled Hymen. Israel*: 219 (2011); based on *Hydnum radula* Fr., *Obs. Mycol*. **2**: 271 (1818); nom. Sanct.

*Type*: [locality unknown, substrate unknown], “*Poria radula*” [Persoon’s hand] (L0117159 [Herb. Ludgd. Bat. 910.277–305] – neotype designated by Donk 1967: 106, as “type”).

**Fig. 5:**
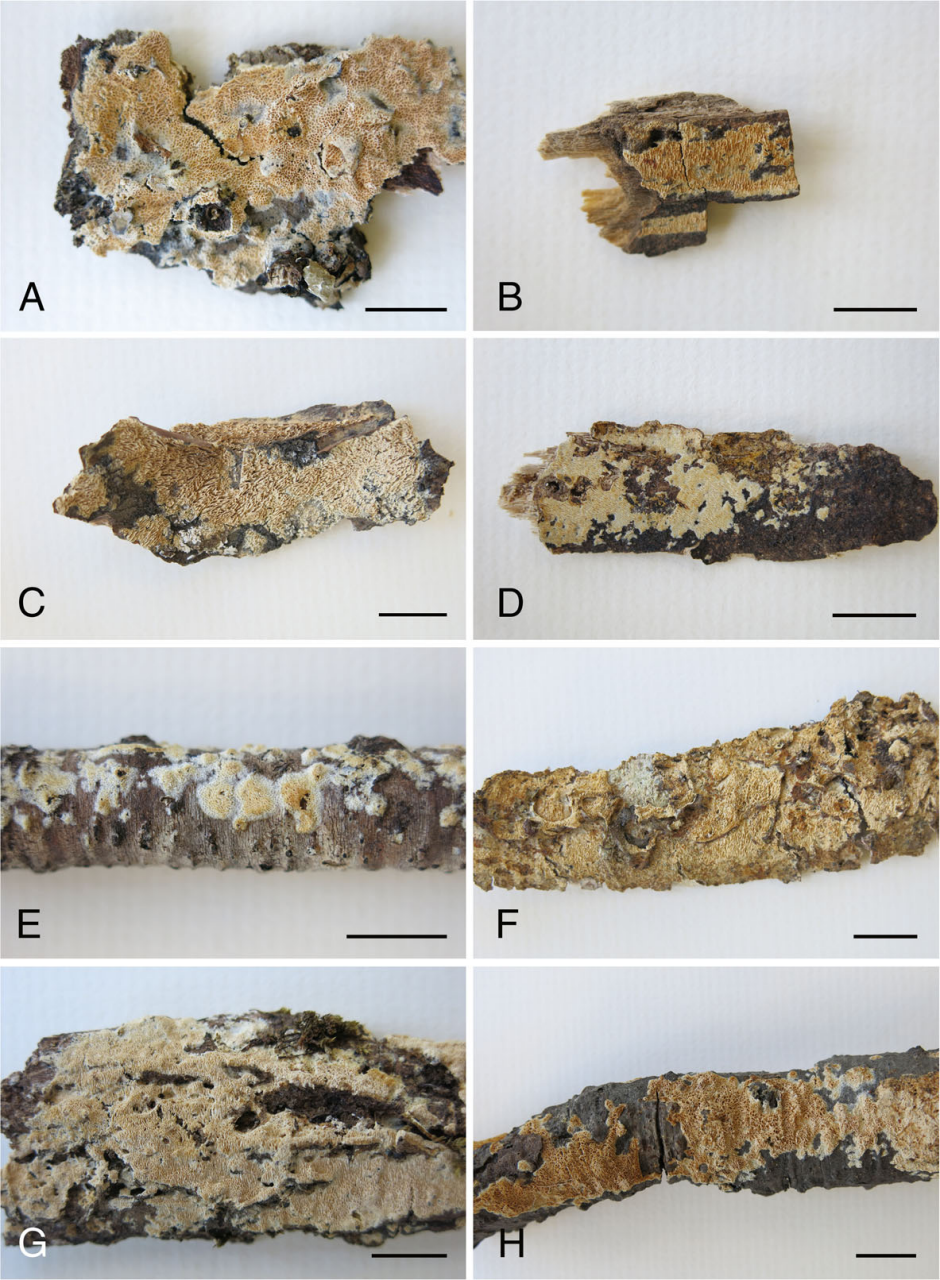
Basidioma of *Xylodon* species. **a**. *Xylodon raduloides* (755MD, MA-Fungi 12,864). **b**, **c**. *Xylodon laurentianus* (B. HHB-719, CFMR, holotype; C. DLL2009–049, CFMR). **d–f**. *Xylodon patagonicus* (D. 19684Tell., MA-Fungi 90,707, holotype; E. 14,180 MD, MA-Fungi 90,702, young specimen; F. 19705Tell., MA-Fungi 90,706, old specimen). **g**, **h.** *Xylodon novozelandicus* (*G. Paulus* 98/20, PDD 70718, holotype; *H. Paulus* 98/104, PDD 70720). Bars = 1 mm

**Fig. 6:**
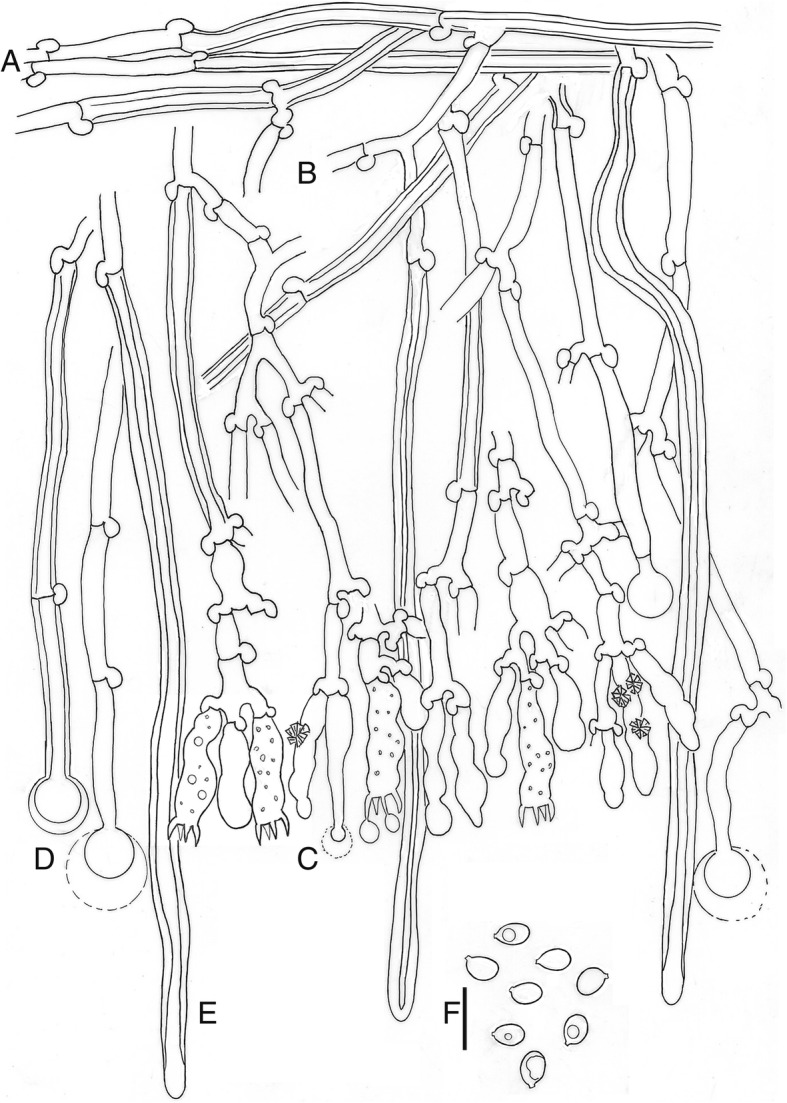
*Xylodon raduloides*, 002JFL, MA-Fungi 90,709. **a.** Subicular hyphae. **b.** Subhymenial hyphae. **c.** Hymenium with cystidia and basidia. **d.** Capitate hyphae. **e.** Tubular hyphae or “skeletocystidia”. **f.** basidiospores. Bar = 10 μm

*Description*: *Basidioma* resupinate, effuse, adnate; hymenophore poroid, 1–4 pores/mm, regular to angular, dissepiments dentate in old specimens, on vertical substrata irregularly irpicoid with flattened teeth, yellow-white to orange-yellow (92. y White – 71. m. OY); margin clearly differentiated, paler (Fig. 5a). *Hyphal system* monomitic; generative hyphae hyaline, thin to thick-walled, sparsely branched, with clamps, 3–5 μm wide; subicular hyphae loosely interwoven, parallel to substratum (Fig. 6a); subhymenial hyphae, perpendicular to the substratum (Fig. 6b). *Cystidia* or rather cystidial elements present: (1) capitate, subcylindrical, fusiform or moniliform cystidia arise from the hymenium (Fig. 6c), sometimes encrusted or with an apical bladder, thin-walled, with basal clamp, 17–26 × 3–5 μm; (2) capitate hyphae arise from the subiculum (Fig. 6d), sometimes with a thin-walled apical bladder, thin to thick-walled, with basal clamp, 30–40 × 3–4.5 μm, apex to 9 μm diam; and (3) tubular hyphae or “skeletocystidia” arise from the subiculum (Fig. 6e), with very thick walls narrowing to the apex, with basal clamp, 120–150 × 3–5 μm. *Basidia* cylindrical to suburniform, (15–)18–21 × 4–5 μm, four sterigmata, with basal clamp (Fig. 6c). *Basidiospores* ellipsoidal, (4.5–)5–5.5(− 7) × (2.5–)3–3.5(− 4.5) μm, hyaline, thin-walled, smooth, guttulate (Fig. 6f). L = 5.34, W = 3.33, Q = 1.60.

*Ecology and habitat*: On rotted wood of *Alnus*, *Carpinus*, *Castanea*, *Eucalyptus*, *Fagus*, *Quercus,* and *Tilia*. Present in areas with seasonal climate, warm and dry summers and cold winters.

*Known distribution*: Widespread in Europe, also known from Africa (Canary Islands and Cameroon).

*Notes*: *Xylodon raduloides* has been reported from northern Iran (Hallenberg, 1983) but we did not study any specimens from the Middle-East region.

The name *X. raduloides* unfortunately had to be introduced because when the combination *Xylodon radula* (Fr.) Tura et al. was made the basionym cited was that of Fries and not that of Persoon, which does not belong to this taxon but to *Basidioradulum radula* (Fr.) Nobles. Even though the name may have been inadvertently misapplied, the combination into *Xylodon* was nevertheless validly published and has to be maintained and applied in accordance with its type, unless a formal conservation proposal was made and eventually accepted. As the name *X. raduloides* has already been introduced to deal with the situation, in order to avoid further possible confusions we retain *X. raduloides* here.

*Material examined*: **Cameroon:** Sakbayeme, 29 Apr. 1980, *Rev. Chas. Schwab,* (NY s.n., as *Schizopora subiculoides*). **– Canary Islands**: *Tenerife*: Esperanza forest, on *Eucalyptus*, 17 Jan. 1990, *R Korf* (MA-Fungi 35,643). **– France**: Moselle, Monterhouse, Canton de Bitche, Forêt Domainale de Monterhouse (parcéle M-43), 280 msl, on dead wood, 25 Oct. 2009, *I. Melo, I. Salcedo & M.T. Telleria 12028IS* (MA-Fungi 79,442); Moselle, Parc Naturel des Vosges du Nord, Pays de Bitche, Forêt Domaniale de Haut III, Rothenbruch Reserve, 49°01′00″N 7°35′50″E, 250 msl, on *Fagus sylvatica*, 29 Oct. 2009, *I. Melo, I. Salcedo & M.T. Telleria 18336Tell.* (MA-Fungi 79,314); Pyrénées-Orientales, Languedoc-Rosillon, Massif des Albères, Lavall, 42°30′27″N 3°00′18″E, 225 msl, on *Quercus suber*, 5 Nov. 2008, *M. Dueñas, I. Melo, I. Salcedo & M.T. Telleria*, *11851IS* (MA-Fungi 78,658); Seine-et-Marne, Fontainebleau, Réserve Integrale, Gorge aux Loups (parcéle 527), 90 msl, on *Fagus sylvatica*, 30 Oct. 2006, *M. Dueñas, I. Melo, I. Salcedo & M.T. Telleria*, *11074MD* (MA-Fungi 70,457). **– Spain:** *Asturias*: Reserva Biológica de Muniellos, on *Quercus robur*, 15 June 1983, *N. Brito, F.D. Calonge, M. Dueñas, V. Pou & M.T. Telleria 755MD* (MA-Fungi 12,864). *Ávila*: Gavilanes, 40°13′18″N 4°50′15″W, on *Quercus ilex*, Nov. 2015, *J. Fernández-López 002JFL* (MA-Fungi 90,709). *Cantabria*: Potes, Monte Tolibe, 450 msl, on *Quercus suber*, 1 Apr. 1985, *P. Coello, M. Dueñas, K. Escalante & M.T. Telleria 6996Tell.* (MA-Fungi 12,877); *Ciudad Real*, Fuencaliente, Robledo de las Ollas, 770 msl, on *Quercus suber*, 12 Apr. 2007, *F. Prieto & A. González GP2291* (MA-Fungi 75,310); ibid., Valle de la Cerceda, 880 msl, on *Quercus pyrenaica*, 16 Dec. 2004, *F. Prieto, A. González & al. GP2162* (MA-Fungi 75,244); ibid., 18 Nov. 2005, *F. Prieto, A. González & al.*, *GP2241* (MA-Fungi 75,130); *Huelva*, El Barraco, Coto de Doñana, on *Quercus suber*, 24 Nov. 1977, *F.D. Calonge* (MA-Fungi 608); *Palencia*, Cervera de Pisuerga, on *Quercus pyrenaica*, 20 Nov. 1984, *N. Brito, M. Dueñas & M.T. Telleria 2266MD* (MA-Fungi 12,778); *Toledo*, between Fresnedilla and El Real de San Vicente, on *Castanea sativa*, 29 May 1988, *M. Dueñas*, *4719MD* (MA-Fungi 22,499); *idem*, *4736MD* (MA-Fungi 22,513); ibid., Velada, los Baldíos, río Guadyerbas, 395 msl, on *Quercus faginea*, 28 Mar. 2006, *F. Prieto, A. González & F.D. Calonge GP2253* (MA-Fungi 75,272).

**Xylodon laurentianus** J. Fernández-López, Telleria, M. Dueñas, & M.P. Martín, **sp. nov.**

**Fig. 7:**
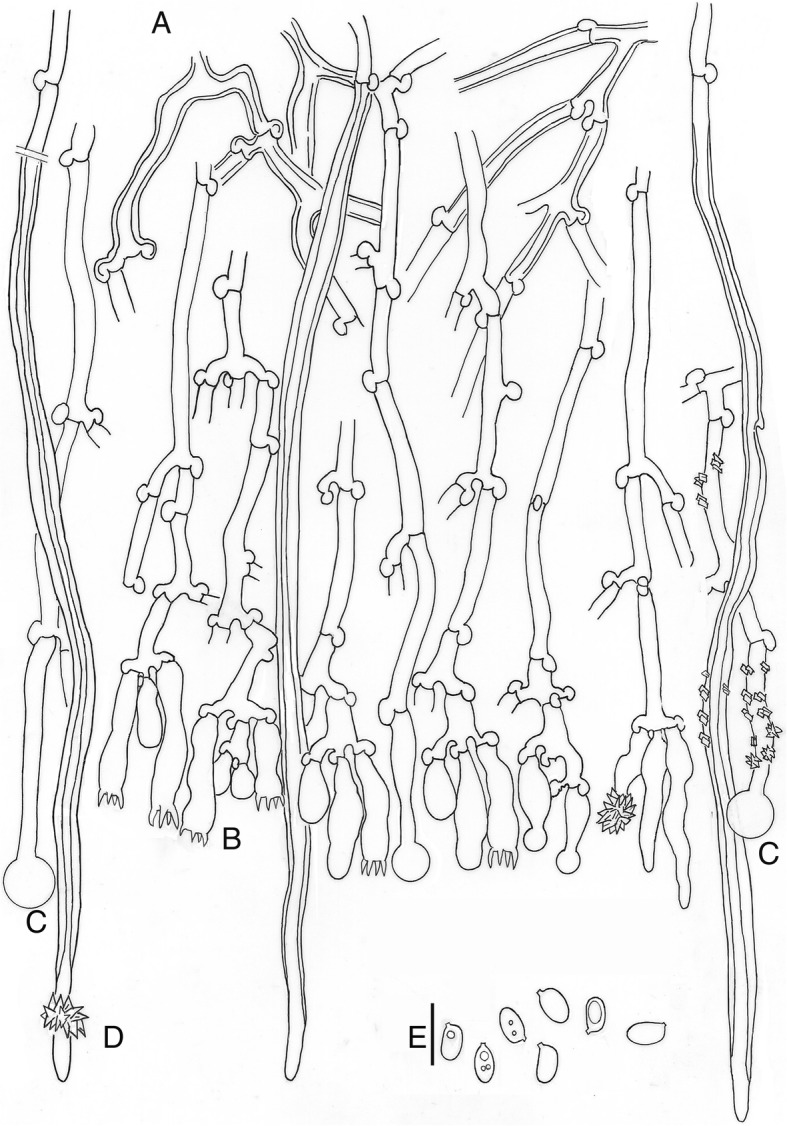
*Xylodon laurentianus*, HHB-719, CFMR, holotype. **a.** Subhymenial hyphae. **b.** Hymenium with cystidia and basidia. **c.** Capitate hyphae. **d.** Tubular hyphae or “skeletocystidia”. **e.** Basidiospores. Bar = 10 μm

MycoBank MB288019. (Figs. 5b, C and 7)

*Etymology*: Named after Laurentia, the ancient geological core (craton) of the North American continent, where the species is distributed.

*Diagnosis*: Morphologically this species is similar to *Xylodon raduloides*, but can be distinguished by the narrowly ellipsoid to subcylindrical basidiospores, (4.5–)5–6 × 2.5–3.5 μm (Q = 1.70), instead of the ellipsoid ones, (4.5–)5–5.5(− 7) × (2.5–)3–3.5(− 4.5) μm (Q = 1.60) in *X*. *raduloides*.

*Type*: USA: *Washington DC*: Ruch Drive, Rock Creek Park, on *Quercus* log, 18 June 1968, *H.H. Burdsall Jr. HHB-719* (CFMR – holotype; Forest Products Laboratory (USDA) – ex-type culture: ITS, LSU and *tef*-1 sequences GenBank KY962845, KY962865, and KY967076).

*Description*: *Basidioma* resupinate, effuse, adnate; hymenophore poroid to labyrinthiform, 1–4 pores/mm, dissepiments lacerate to dentate in old specimens, on vertical substrata irregularly irpicoid with flattened teeth, orange-yellow (70. l. OY – 71. m. OY); margin not clearly differentiated. *Hyphal system* monomitic; generative hyphae hyaline, thin to thick-walled, sparsely branched, with clamps, 3–5 μm wide; subicular hyphae not seen; subhymenial hyphae loosely interwoven, perpendicular to substratum. *Cystidia* or rather cystidial elements present: (1) capitate and subulate cystidia, sometimes encrusted, arise from the hymenium, thin-walled, with basal clamp, 14–32 × 3.5–5 μm; (2) capitate hyphae sometimes encrusted, thin-walled, with basal clamp, 25–46 × 3–4 μm, apex to 8 μm diam; and (3) tubular hyphae or “skeletocystidia” sometimes encrusted, with very thick walls narrowing to the apex, with basal clamp, 170–200 × 3.5–5.5 μm. *Basidia* cylindrical to suburniform, (13–)18–26 × 4.5–5.5 μm, four sterigmata, with basal clamp. *Basidiospores* narrowly ellipsoidal to subcylindrical, (4.5–)5–6 × 2.5–3.5 μm, hyaline, thin-walled, smooth, guttulate. L = 5.22, W = 3.08, Q = 1.70.

*Ecology and habitat*: On dead wood of *Quercus*. Present in areas with a seasonal climate, warm and dry summers and cold winters.

*Known distribution*: Reported from Central and Eastern USA (Minnesota and Washington DC).

*Additional material examined*: USA: *Minnesota*: St Louis County, Independence, on dead wood, 28 Oct. 2009, *D.L. Lindner DLL2009–049* (CFMR).

**Xylodon patagonicus** J. Fernández-López, Telleria, M. Dueñas, & M.P. Martín, **sp. nov.**

**Fig. 8:**
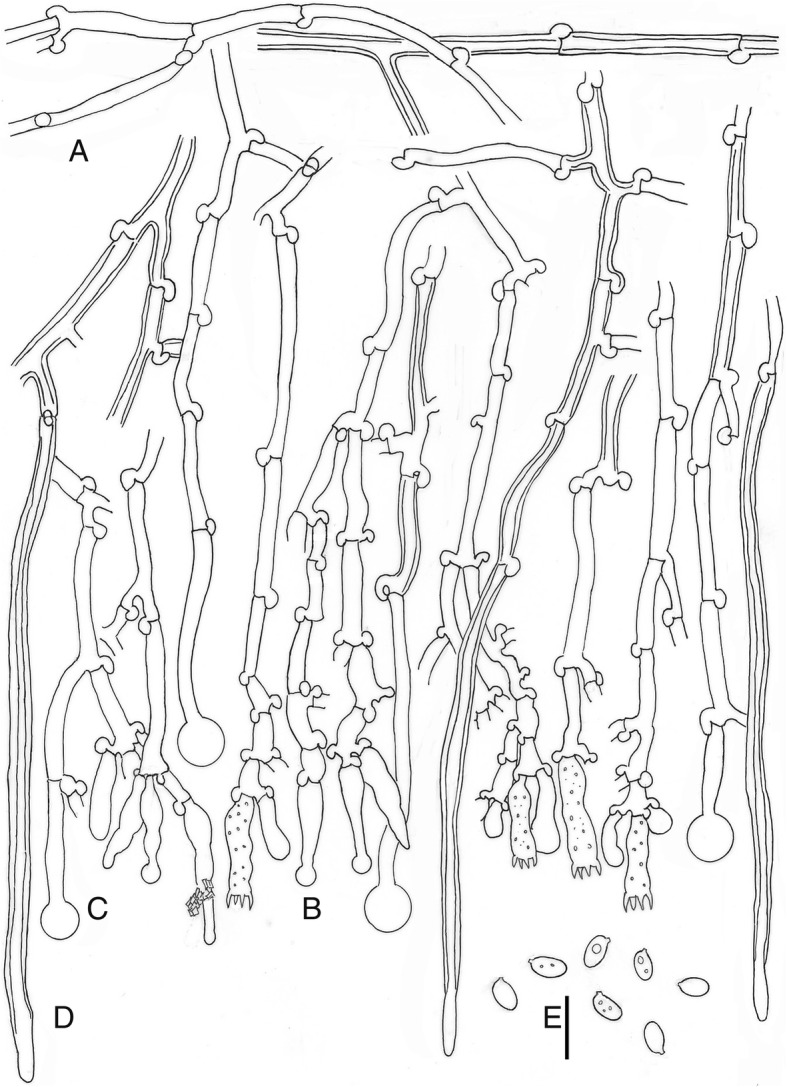
*Xylodon patagonicus*, 19684Tell., MA-Fungi 90,707, holotype. **a.** Subhymenial hyphae. **b.** Hymenium with cystidia and basidia. **c.** Capitate hyphae. **d.** Tubular hyphae or “skeletocystidia”. **e.** Basidiospores. Bar = 10 μm

MycoBank MB288018. (Figs. 5d-f and 8)

*Etymology*. Named after Patagonia, the region where the holotype was collected.

*Diagnosis*: Morphologically similar to *Xylodon raduloides*, but differs in having smaller basidia, 13–18 × 3–4.5 μm, and shorter basidiospores, (3.5–)4–5.5(− 6) × (2–)2.5–3.5(− 4.5) μm with Q = 1.46.

*Type*: **Chile**: *Los Lagos* (*X Región*): Palena, Comuna Hualaihué, Comau Fjord, Huinay, “Cementerio de Alerces”, 42°21′57.9″S 72°24′56.9″W, 30 msl, on *Amomyrtus luma*, 29 Apr. 2012, *M. Dueñas, M.P. Martín & M.T. Telleria 19684Tell* (MA-Fungi 90,707 –holotype; ITS, LSU and *rpb*2 sequences GenBank KY962837, KY962855, and KY967061).

*Description*: *Basidioma* resupinate, effuse, adnate, orbicular to confluent; hymenophore poroid to labyrinthiform, 1–5 pores/mm, dissepiments lacerate to dentate in old specimens, on vertical substrata irregularly irpicoid with flattened teeth, yellow-white to orange-yellow (92. y White – 71. m. OY); margin not clearly differentiated. *Hyphal system* monomitic; generative hyphae hyaline, thin to thick-walled, branched, with clamps, 2.5–4 μm wide; subiculum not clearly differentiated; subhymenial hyphae loosely interwoven, perpendicular to substratum. *Cystidia* or rather cystidial elements present: (1) capitate and subulate cystidia, sometimes encrusted, arise from the hymenium, thin-walled, with basal clamp, 17.5–25 × 3–4 μm; (2) capitate hyphae, thin-walled, basal clamp, 22–56 × 2.5–4 μm, apex up to 8 μm diam; and (3) tubular hyphae or “skeletocystidia” very thick-walled narrowing to the apex, basal clamp, 80–115 × 3–4 μm. *Basidia* cylindrical to suburniform, 13–18 × 3–4.5 μm, four sterigmata, with basal clamp. *Basidiospores* ellipsoidal, (3.5)4–5.5(− 6) × (2–)2.5–3.5(− 4.5) μm, hyaline, thin-walled, smooth, guttulate. L = 4.56, W = 3.11, Q = 1.46.

*Ecology and habitat*: On dead wood of *Nothofagus nitida* and *N. dombeyi* (*Nothofagaceae*), and *Amomyrtus luma* (*Myrtaceae*). Present in areas with a mild climate, with low annual variations in temperature, and high humidity during dry season.

*Known distribution*: Reported from the Patagonian region (southern Chile and southern Argentina).

*Notes*: *Poria platensis* was described from Argentina by Spegazzini (1902) and later synonymized by Lowe (1963) with *Schizopora paradoxa*. Hallenberg (1983) segregated *Schizopora radula* (i.e. *Xylodon raduloides*) from *S. paradoxa* and therefore *P. platensis* could be related to the *X. raduloides* complex, and more specifically with *X. patagonicus,* but the substrate was given as on *Pinus* beams rather than on a hardwood so is unlikely to be conspecific. No specimens of this taxon were available to study and investigate this further.

*Additional material examined*: **Chile**: *Los Lagos* (*X Región*): Palena, Comau Fjord, Comuna Hualaihué, Huinay, path to Cerro del Tambor, 42°22′53.2″S 72°24′44.0″W, 125 msl, on fallen logs, 26 Apr. 2012*, M. Dueñas, M.P. Martín & M.T. Telleria 3341MPM* (MA-Fungi 90,704); *idem*, *3340MPM* (MA-Fungi 90,708); ibid., on *Nothofagus nitida* wood, 26 Apr. 2012, *M. Dueñas, M.P. Martín & M.T. Telleria 14007MD* (MA-Fungi 90,705); ibid., path to Cerro del Tambor behind hydroelectric power station, 42°22′54.2″S 72°24′53.5″W, 202 msl, on fallen logs, 8 May 2013, *M. Dueñas, M.P. Martín & M.T. Telleria 3567MPM* (MA-Fungi 90,703); ibid., “Derrumbe Antiguo”, 42°22′17.0″S 72°24′12.2″W, 120 msl, on *Nothofagus dombeyi*, 1 May 2012, *M. Dueñas, M.P. Martín & M.T. Telleria 14,180 MD* (MA-Fungi 90,702); ibid., Lloncochaigua river bank, near to the bridge, 42°22′09.0″S 72°24′42.7″W), 19 msl, on dead wood, 30 Apr. 2012, *M. Dueñas, M.P. Martín & M.T. Telleria 19705Tell* (MA-Fungi 90,706).

**Xylodon novozelandicus** J. Fernández-López, Telleria, M. Dueñas, & M.P. Martín, **sp. nov.**

**Fig. 9:**
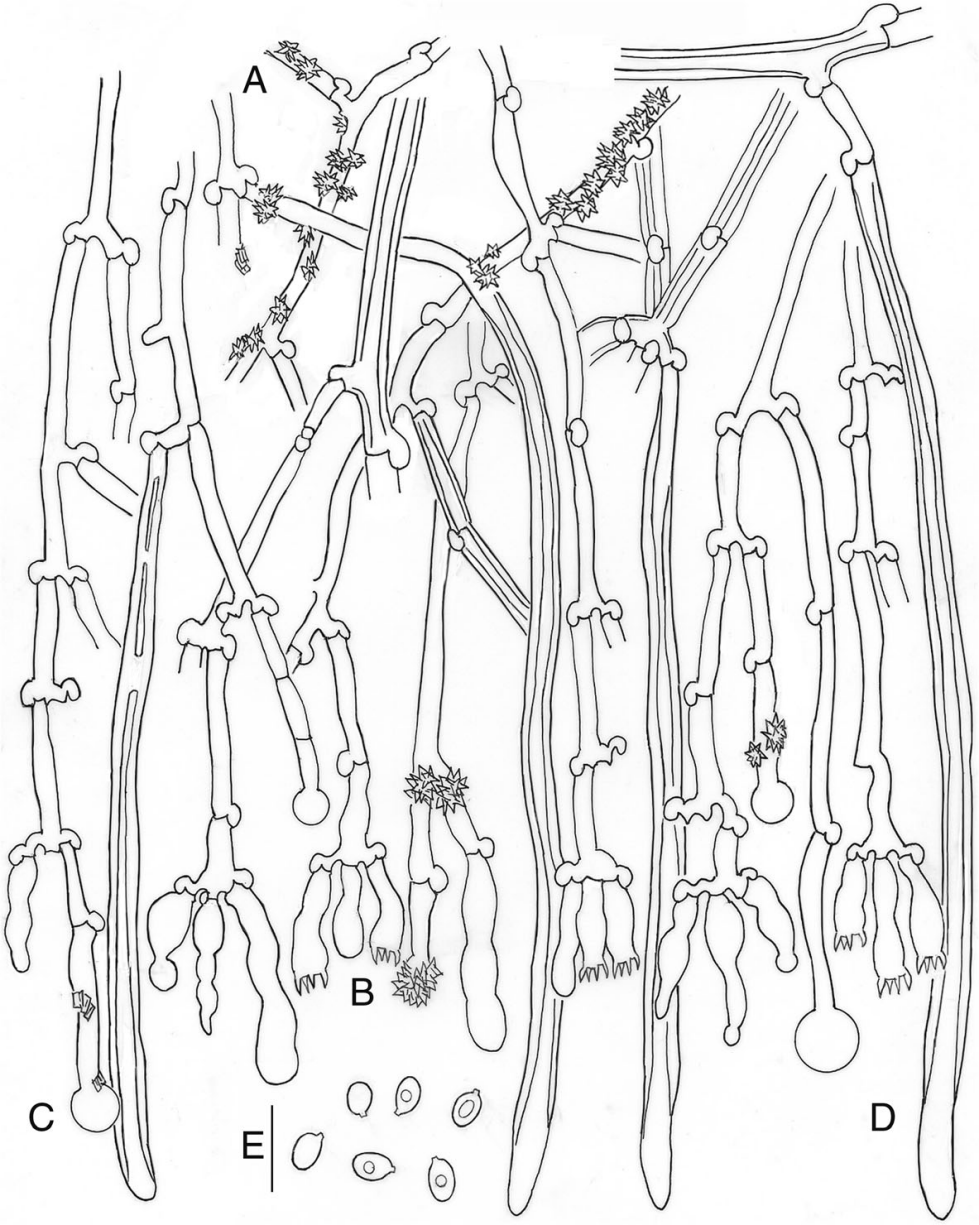
*Xylodon novozelandicus*, Paulus 98/20, PDD 70718, holotype. **a.** Subhymenial hyphae. **b.** Hymenium with cystidia and basidia. **c.** Capitate hyphae. **d.** Tubular hyphae or “skeletocystidia”. **e.** Basidiospores. Bar = 10 μm

MycoBank MB828020. (Figs. 5g, h and 9)

*Etymology*: Named after New Zealand, where the holotype was collected.

*Diagnosis*: This species is morphologically similar to *Xylodon patagonicus*, but differs in having subcylindrical cystidia arising from the hymenium.

*Type:* **New Zealand**: Wellington, Kaitoki, Swingbridge Track, on dead wood in “Podocarp/Broadleaf forest”, 22 Feb. 1998, *I.G. Steer & B.C. Paulus*, *B.C. Paulus 98/20* (PDD 70718 – holotype; ICMP13838 – ex-type culture; ITS, LSU and *tef*-1 sequences GenBank AF145578, KY962851, and KY967069).

*Description: Basidioma* resupinate, effuse, adnate; hymenophore poroid to labyrinthiform, 1–4 pores/mm, dissepiments lacerate to dentate in old specimens, orange-yellow (70. l. OY); margin not clearly differentiated. *Hyphal system* monomitic; generative hyphae hyaline, thin to thick-walled, encrusted, branched, with clamps, 2.5–4.5 μm wide; subicular hyphae not seen; subhymenial hyphae loosely interwoven, perpendicular to the substratum. *Cystidia* or rather cystidial elements present: (1) capitate, subcylindrical and subulate cystidia, sometimes encrusted, arise from the hymenium, thin-walled, basal clamped, 13–27 × 3.5–4.5 μm; (2) capitate hyphae, thin-walled, with basal clamp, 28–34 × 2–3.5 μm, apex up to 9 μm diam; and (3) tubular hyphae or “skeletocystidia” very thick-walled narrowing to the apex, with basal clamp, 110–150 × 3.5–4.5 μm. *Basidia* cylindrical to suburniform, (9–)10–15 × 3–4.5 μm, four sterigmata, with basal clamp. *Basidiospores* ellipsoidal, 4–6 × (2.5–)3–4(− 4.5) μm, hyaline, thin-walled, smooth, guttulate. L = 4.71, W = 3.21, Q = 1.47.

*Ecology and habitat*: Growing on dead wood identified as *Carpinus betulus* and *Fuscospora cliffortioides*. Present in areas with a mild climate, low annual variations in temperature, and high humidity during the dry season.

*Known distribution*: Reported from Australasia (Australia and New Zealand). Also found in France and Canada (British Columbia).

*Notes*: Timber trade and transport could easily explain this distribution pattern for both French and Canadian samples (Table 1, Fig. 3). No morphological study was carried out on the Canadian sample, since the ITS sequence was obtained from a culture. However, a morphological study of the French specimen revealed differences in spore morphology from New Zealand material, being similar to those from the European clade, so this trait could be linked to environmental conditions.

*Additional material examined*: **France**: *Côtes-d’Armor*: Commune de Plévenon, Saint Cast-le Guildo, La Fresnaye, 48°38′33.7″N, 2°16′31.7″W, 15 msl, on *Carpinus betulus* wood, 24 Oct. 2010, *M. Dueñas, I. Melo, I. Salcedo & M.T. Telleria 12836IS* (MA-Fungi 74,919). **– New Zealand**: Buller, South of Punakaiki Field Centre, “Lifestyle Blocks”, on fallen branches, 16 May 1998, *I.G. Steer & B.C. Paulus*, *B.C. Paulus 98/81* (PDD 70716; ICMP 13841 – culture); Mid-Canterbury, on *Fuscospora cliffortioides* wood, 11 May 2006, *A. Roberts & B.C. Paulus* (PDD 91616); ibid., Christchurch, Riccarton Bush, fallen branch, 17 May 1998, *I.G. Steer & B.C. Paulus*, *B.C. Paulus 98/104* (PDD 70720; ICMP 13840 – culture).

## DISCUSSION

Morphological species recognition has limits in a group like the *Xylodon raduloides* species complex; alone, it would not likely identify the species and geographic diversity revealed during this study. Our ITS and LSU analyses revealed four species within the *X*. *raduloides* complex (Fig. 2). These species were confirmed in a multi-locus coalescent framework, since the Bayes factors approach further established the ITS species proposal (hypothesis-C) as the most probable given the data. The ability of ITS sequence data to detect hidden diversity in fungi has been questioned leading to the suggestion that a multi-locus approach should be used (Balasundaram et al. 2015). Our results suggest that the ITS region performs well for the *X. raduloides* species complex, but this is likely context dependent so general inferences about its utility in species delimitation should be cautious as they may strongly depend on the group being studied (Balasundaram et al. 2015, Wilson et al. 2017a).

Although the ITS region is a powerful tool for discriminating between fungal species, as it is a non-transcribed, non-coding region makes it prone to accumulate homoplasies (Nagy et al. 2012). This accumulation of random homoplasies means the ITS region alone is not generally useful to study inter-species relationships. Thus, the multi-locus species coalescent approach allowed for comparison of relationships between species since internal nodes showed high support for geographic relationships (Fig. 3). Salgado-Salazar et al. (2013) used this method to separate 16 distinct highly supported lineages in the morphologically circumscribed *Thelonectria discophora* which were linked to different geographic areas and ecological settings.

The multi-locus phylogenetic approach, which incorporated *rpb*2, and *tef*-1α along with ITS and LSU sequence data, revealed that Holarctic taxa (*X*. *raduloides* and *X*. *laurentianus*) were more genetically related, and that *X. patagonicus* and *X*. *novozelandicus* shared a recent common ancestor (Fig. 3). While it has been demonstrated that fungal distributions can be human-mediated (Fuller et al. 2013), only two specific instances of possible human translocation are supported by our results: specimens of *X*. *novozelandicus* were reported from France and Canada. As a result, the extant biogeographic distribution of the *Xylodon* species studied is likely due to natural processes.

Our results correlate with geography and suggest allopatric differentiation within the *Xylodon* species in this study, confirming the proposal of Paulus et al. (2000). This geographic phylogenetic structure has been observed in other basidiomycetes, like the *Schizophyllum commune* complex (James et al. 2001), the genus *Lentinula* (Hibbett 2001) and the lethal amanitas (*Amanita* sect. *Phalloideae*; Cai et al. 2014). This reveals the importance of geographic separation in genetic isolation and gene flow in fungi (Taylor et al. 2006). In other cases, such as in *Laccaria* (ectomycorrhizal fungi), in addition to geographic barriers, a study of host associations is necessary to obtain a proper understanding of the factors that explain species distributions (Wilson et al. 2017b). These results show that multiple factors can affect the biogeographical patterns in fungi.

Biogeographical patterns shown by the Southern Hemisphere species agree with the general pattern observed for most Gondwanan groups of plants (Sanmartín & Ronquist 2004). A deep vicariance event could be inferred between *X. patagonicus* and *X*. *novozelandicus*, which could be due to the geological breakup of the supercontinent Gondwana approximately 80 MYA (Scotese et al. 1988). Molecular differences were not found between Australian and New Zealand specimens of *X*. *novozelandicus*, suggesting the absence of genetic isolation. Dispersal events between Australia and New Zealand may well explain how a single species in these two areas is maintained, while remaining genetically isolated from *X. patagonicus*. This dispersal ability has been commonly observed in Southern Hemisphere plants (Seberg 1991, Linder & Crisp 1995, Knapp et al. 2005) and fungi (Moncalvo & Buchanan 2008, Peterson et al. 2010, Wilson et al. 2017b).

The close relationship between woody plants and corticioid fungi suggests a shared historical biogeography. In this context, little is known about host specificity for the *X*. *raduloides* complex. A variety of hosts (*Alnus*, *Carpinus, Quercus*, *Tilia*, etc.) have been reported in the European region (Langer 1994, Ryvarden & Melo 2014), while for the New Zealand region it has been reported on decayed southern beech: *Fuscospora cliffortioides*, *F. fusca*, *Lophozonia menziesii* (Clinton et al. 2009). In general, current knowledge points toward a broad range of hosts for the *X*. *raduloides* complex, which could account for the worldwide distribution.

The geographic and phylogenetic patterns confirm that for the *X*. *raduloides* complex, as in other basidiomycetes (Hibbett 2001), the hypothesis “everything is everywhere” is not applicable. The traditional dependence on morphological species recognition criteria has led to an underestimate of species diversity and did not reveal the actual distribution patterns for the *Xylodon* species in our study.

Morphological analysis of diversity in the *X*. *raduloides* complex confirms that basidiospore morphology may be only partially effective as an inter-specific diagnostic character in these fungi. Spore shape (length to width ratio, Q) was able to distinguish between Northern and Southern Hemisphere groups (Fig. 4). Northern Hemisphere specimens have longer spores, while spores of Southern Hemisphere species have a more spherical shape. No statistical differences were found among intra-hemisphere specimens (Fig. 4). This observation could be due to the close phylogenetic relationship within Northern and Southern Hemisphere species, supported by our molecular results (Figs. 2, 3). While little attention has been paid to spore morphology (Parmasto & Parmasto 1992), their importance as dispersion propagules, in sexual reproduction and gene flow in fungi (Kauserud et al. 2008) makes them an informative diagnostic character even when morphological stasis is observed for other traits. However, spore morphology may be insufficient to discriminate recent speciation in the inter-Hemisphere *X*. *raduloides* complex.

Environmental niche analyses performed for the species complex showed non-equivalence between Northern and Southern Hemisphere species niches (Fig. 3). Bioclimatic associations also were in concordance with molecular data and separate the complex by Hemisphere. Environmental traits that defined these two groups could be summarized in an isothermal-seasonal gradient. Northern Hemisphere species are acclimated to a more seasonal environment, with warmer and drier summers and colder winters; while Southern Hemisphere species fructified in mild climates, characterized by low annual thermal variations and more humidity during the dry season. These results indicate that phylogenetically related species occupy similar environmental niches.

The correlation between spore morphology and environmental features in the *X*. *raduloides* complex is interesting. There is a demonstrated relationship between spore morphology and environmental conditions in many other fungi (Kauserud et al. 2008). The metabolic costs of spore production make it subject to evolutionary fitness (Stearns 1992). Larger spores are correlated with more seasonal areas; this association could be explained by the necessity of storing more nutrients for the transition from dry season to sporophore production (Kauserud et al. 2011). This hypothesis is in agreement with our results for the *X*. *raduloides* complex, since the Northern Hemisphere group –with a more seasonal climate– showed spores of greater volume by virtue of being longer than the spores from the Southern Hemisphere species (Fig. 4). Whether or not the concordance between environmental characteristics and spore morphology is indicative of a cause and effect relationship remains to be evaluated in this complex, since the correlation between environmental and morphological traits could also be explained by the shared evolutionary and geographic history between sister species. Further phylogenetic comparative analysis, e.g. assessing the phylogenetic signal for spore morphology or environmental preferences (Felsenstein 1985, Grafen 1989, Revell et al. 2008) should be conducted to specifically test these hypotheses.

## Conclusions

In this study, the diversity of *Xylodon raduloides* is addressed from multiple complementary perspectives, separating this species complex into four species: *Xylodon raduloides*, *X. laurentianus* sp. nov., *X. novozelandicus* sp. nov., and *X. patagonicus* sp. nov.. These species are distributed geographically in Europe, North America, Australia–New Zealand and Patagonia, respectively. The methods of integrative taxonomy, using molecular, morphological and ecological traits, demonstrates that molecular results are confirmed by morphological and ecological traits that could be used as diagnostic characters for other species complexes of corticioid fungi. The combination of molecular tools with morphological and ecological approaches could also clarify what traits have been affected by phylogenetic constraints, or those that are driving the evolutionary processes.

## Data Availability

All material examined are located in public fungaria and culture collections. All sequences files are available from the GenBank database. The complete list of accession numbers is included in Table 1. They will be public after the paper is published.

## Abbreviations

ANOVA: Analysis of Variance
BFD: Bayes factor delimitation
BI: Bayesian inference
BIC: Bayesian information criterion
CFMR: Center for Forest Mycology Research
D index: Distance index
DDBJ: DNA Data Bank of Japan
DNA: Deoxyribonucleic Acid
EMBL: The European Molecular Biology Laboratory
ESS: Effective Simple Size
GCPSR: Genealogical Concordance Phylogenetic Species Recognition
GIS: Geographic Information System
HKY + G: Hasegawa-Kishino-Yano plus Gamma
HSD: Honestly-significant-difference
ICMP: International Collection of Microorganisms for Plants
ISCC-NBS: Inter-Society Color Council-National Bureau for Standards
ITS: Internal Transcribed Spacer
L: Length
LSU: Large Subunit
MA-Fungi: Fungus collections of the Real Jardín Botánico de Madrid, Spain
MCMC: Markov chain Monte Carlo
MLEs: Marginal Likelihood for Each hypothesis
MYA: Million Years Ago
nrDNA: nuclear ribosomal DNA
NY: The William and Lynda Steere Herbarium of the New York Botanical Garden
PC: Principal Components
PCA: Principal Components Analysis
PDD: New Zealand Fungarium
PP: Posterior Probabilities
*P*-value: Probability value
Q: Length to width ratio
Q-Q plot: Qualitative-Qualitative plot
R: A language and environment for statistical computing and graphics
rpb2: the second-largest subunit of RNA polymerase II
tef-1α: Translation elongation factor 1 α
USDA: United States Department of Agriculture
vs: Versus
W: Width
